# Differential Privacy via Haar Wavelet Transform and Gaussian Mechanism for Range Query

**DOI:** 10.1155/2022/8139813

**Published:** 2022-09-12

**Authors:** Dong Chen, Yanjuan Li, Jiaquan Chen, Hongbo Bi, Xiajun Ding

**Affiliations:** College of Electrical and Information Engineering, Quzhou University, Quzhou 324000, China

## Abstract

Range query is the hot topic of the privacy-preserving data publishing. To preserve privacy, the large range query means more accumulate noise will be injected into the input data. This study presents a research on differential privacy for range query via Haar wavelet transform and Gaussian mechanism. First, the noise injected into the input data via Laplace mechanism is analyzed, and we conclude that it is difficult to judge the level of privacy protection based on the Haar wavelet transform and Laplace mechanism for range query because the sum of independent random Laplace variables is not a variable of a Laplace distribution. Second, the method of injecting noise into Haar wavelet coefficients via Gaussian mechanism is proposed in this study. Finally, the maximum variance for any range query under the framework of Haar wavelet transform and Gaussian mechanism is given. The analysis shows that using Haar wavelet transform and Gaussian mechanism, we can preserve the differential privacy for each input data and any range query, and the variance of noise is far less than that just using the Gaussian mechanism. In an experimental study on the dataset age extracted from IPUM's census data of the United States, we confirm that the proposed mechanism has much smaller maximum variance of noises than the Gaussian mechanism for range-count queries.

## 1. Introduction

Over the past ten years, differential privacy has become one of the important methods in the area of privacy-preserving for statistical databases. Differential privacy is a promising scheme for publishing statistical query results of sensitive data, which has a strong privacy guarantee for opponents with arbitrary background knowledge [1–6]. The strong privacy guarantee of differential privacy ensures that any individuals in the data set will not significantly affect the analysis results of the data set. At present, three basic mechanisms are widely used to ensure differential privacy: Laplace mechanism, Gaussian mechanism, and exponential mechanism. Laplacian and Gaussian mechanisms are applicable to numerical queries, and exponential mechanisms are applicable to non-numeric queries [7–9]. Recently, differential privacy is adopted on many research field, such as social network publishing [10–12], crowdsourced data publication [13, 14], and genomic privacy [15–17].

Along with a long-range query scope, the accumulation of noise in the range query answered for privacy preserving can affect the usability of the released data [18, 19]. To reduce the accumulation of noise, the method of hierarchical decompositions is usually employed [20]. Zhang et al. proposed a differentially private algorithm for hierarchical decompositions and named it as PrivTree. This histogram construction algorithm eliminates the dependency on a predefined limit parameter. The privacy-preserving range query is adopted in the field of Internet of Things (IoT) in recent years [21–23]. Cai et al. studied the transaction approximate range counting problem of large IoT data. They proposed a sampling-based method to generate approximate counting results. For privacy reasons, these results will be further disturbed and then published. It is theoretically proved that this result achieves unbiasedness, bounded variance and enhances privacy guarantee under differential privacy. Mahdikhani et al. proposed a communication efficient privacy protection range query in the fog-enhanced Internet of things. The feature of this scheme is that it adopts the Paillier homomorphic cryptosystem and the ingenious bloom filter data structure to achieve better privacy and higher count aggregation efficiency in the range query scenario of protecting privacy. Histogram is a representative and popular tool for data publishing and visualization tasks. Nowadays, protecting private data and preventing the leakage of sensitive information have become one of the main challenges faced by histogram [24–26]. Histogram is the result of a group of counting queries. It is the core statistical tool for reporting data distribution. In fact, it is regarded as the basic method of many other statistical analyses, such as range query [27]. The advantage of histogram representation is that it limits the sensitivity to noise. For example, when histograms are used to support range or count queries, adding or deleting a single record will affect at most one box. Therefore, the sensitivity of range or count query on the histogram is equal to 1, and the amount of additional noise per box will be relatively small [28]. For the differential privacy of long-range queries on the histogram, the accumulation of noise is a key issue that needs to be focused.

Discrete wavelet transform (DWT) is an important technology in signal and image processing [29–31]. Lifting scheme, also called second generation wavelet, has many advantages comparing with the first generation wavelet, such as in-place computation, integer-to-integer transforms, and speed [32–34]. Wavelet-based privacy preserving is studied in recent years [35–37]. Xiao et al. propose the differential privacy via Haar wavelet transform. They introduce a data publishing technique named Privelet. Privelet not only ensures *ε*-differential privacy but also provides accurate results for range query by injecting less noise into wavelet coefficients. The mechanism that can be used to build the differential privacy in Privelet is Laplace distribution. The Laplace mechanism, which is used to guarantee differential privacy in Privelet, maybe not a good choice for building the privacy-preserving system based on discrete wavelet. The reason is that the Laplace noise does not have the property of additivity. That is, the sum of two Laplace distributions is not a Laplace distribution. That means we cannot obtain an analyzable noise distribution by wavelet reconstruction where the Laplace noise is injected into the wavelet coefficients.

The Gaussian mechanism for differential privacy is proposed by Dwork [38, 39]. The Gaussian noise can be used in the structuring of hierarchical decompositions, such as wavelet transforms. The property of additivity of Gaussian noise is very important for the reconstruction of noise data. On the one hand, additivity can ensure that the reconstructed noise is still Gaussian noise; on the other hand, some noise can be eliminated during reconstruction.

In view of the above analysis, we will do some research on differential privacy via Gaussian mechanism and lifting scheme of Haar wavelet transform for range query in this study. In summary, the main contributions of this work are as follows:

1. Differential privacy using lifting Haar wavelet transform and Laplace mechanism is analyzed in this study. The distribution of noise injected into the input data via wavelet reconstruction is discussed and we conclude that they are not noise of Laplace distribution.
2. Differential privacy based on lifting Haar wavelet transform and Gaussian mechanism is constructed in this study. For range query, our analysis shows that the noise actually added into a certain range of original data is much less than the sum of noise at each data for the proposed mechanism.
3. Differential privacy for range query via lifting Haar wavelet and Guassian mechanism is discussed. We give an algorithm to compute the maximum variance of any range query for any given parameter *l* (suppose the length of input data is 2^*l*^). Moreover, we give a coarse estimation of the maximum variance of range query using a function expression. Finally, we give an experimental study using the proposed mechanism, and the results show the proposed mechanism has a much smaller maximum variance of noise than the Gaussian mechanism for range query.

The remainder of the study is organized as follows: Section 2 introduces the fundamental definitions and theorems about the differential privacy and its implement mechanism.  Section 3 gives the theorems for how to inject Gaussian noise into the Haar wavelet coefficients.  Section 4 analysis the noise of range query under the framework of Gaussian mechanism and Haar wavelet. First, the computing method for the variance of range query is given. Second, the algorithm of computing maximum variance for any range query is introduced, and how to get the interval of the range query when obtaining the maximum variance is introduced in detail. Finally, the coarse estimation of the maximum variance of range query is given as a function expression.  Section 5 introduces the experimental verification of the computing of maximum variance for the range query based on Gaussian mechanism and lifting Haar wavelet. Conclusions is given in Section 6.

## 2. Preliminaries

In this section, the fundamental definitions and theorems about the differential privacy and its implement mechanism are introduced first. Furthermore, the method of injecting Laplace noise into the Haar wavelet coefficients is given. They are the basis of the other sections.

### 2.1. Differential Privacy

**Definition 1 ((*ε*, *δ*)-Differential privacy [38, 39]).**

A randomized mechanism *M* with domain *ℕ*^|*χ*|^⟶*ℝ*^*d*^ is (*ε*, *δ*)-differential privacy if for all *S*⊆*Range*(*M*) and for all *x*, *y* ∈ *ℕ*^|*χ*|^ such that ‖*x* − *y*‖_1_ ≤ 1.

(1) $$\begin{array}{c} \mathrm{Pr}\;\left[M\left(x\right)\in S\right]\leq \exp\left(\varepsilon \right)\mathrm{Pr}\;\left[M\left(y\right)\in S\right]+\delta \text{,} \end{array}$$

where the symbol ‖*x*‖_1_ denotes the *ℓ*_1_-norm of a database *x*, ‖*x*‖_1_=∑|*x*_*i*_|, and ‖*x* − *y*‖_1_ denotes the *ℓ*_1_-distance between two databases *x* and *y*.

### 2.2. Laplace Mechanism

**Definition 2 (*ℓ*_1_-sensitivity [39]).**

Let *ℕ*^|*χ*|^⟶*ℝ*^*d*^ be an arbitrary *d*-dimensional function, then define the *ℓ*_1_-sensitivity of function *f* as follows:

(2) $$\begin{array}{c} \Delta_{1}f=\max_{\begin{array}{c} x,y\in N^{\left|\chi \right|}\\ {\left\|x-y\right\|}_{1}=1 \end{array}}{\left\|f\left(x\right)-f\left(y\right)\right\|}_{1}\text{.} \end{array}$$

**Definition 3 .**

(Laplace distribution, *Lap*(*λ*)). The Laplace distribution with mean zero and scale *λ* is the distribution with probability density function:

(3) $$\begin{array}{c} Lap\left(x|\lambda \right)=\left(\frac{1}{2\lambda }\right)\exp\;\left(-\frac{\left|x\right|}{\lambda }\right)\text{.} \end{array}$$

In Definition 3, the variance of this distribution is *σ*^2^=2*λ*^2^. We write *Lap*(*λ*) to denote the Laplace distribution with mean zero and scale *λ* in this study.

**Theorem 1 (Laplace mechanism [39]).**

Let *f* is a function with *ℓ*_1_-sensitivity, the Laplace mechanism, which adds independently random drawn noise distributed as *Lap*(Δ_1_*f*/*ε*) into each of the *d* components of the output, preserves (*ε*, 0)-differential privacy.

**Remark 1 .**

Throughout the study, we use the term “noise” to refer to a random variable with a zero mean.

### 2.3. Gaussian Mechanism

**Definition 4 (*ℓ*_2_-sensitivity [39]).**

Let *f* : *ℕ*^|*χ*|^⟶*ℝ*^*d*^ be an arbitrary *d*-dimensional function, then define the *ℓ*_2_-sensitivity of function *f* as follows:

(4) $$\begin{array}{c} \Delta_{2}f=\max_{\begin{array}{c} x,y\in N^{\left|\chi \right|}\\ {\left\|x-y\right\|}_{2}=1 \end{array}}{\left\|f\left(x\right)-f\left(y\right)\right\|}_{2}\text{,} \end{array}$$

where the symbol ‖*x*‖_2_ denotes the *ℓ*_2_-norm of a database *x*, ‖*x*‖_2_=∑*x*_*i*_^2^, and ‖*f*(*x*) − *f*(*y*)‖_2_ denotes the *ℓ*_2_-distance between *f*(*x*) and *f*(*y*).

**Definition 5 (Gaussian distribution, Gauss(*σ*^2^)).**

The Gaussian distribution with mean zero and variance *σ*^2^ is the distribution with probability density function:

(5) $$\begin{array}{c} Gauss\left(x|\sigma^{2}\right)=\frac{1}{\left(\sqrt{2\pi }\sigma \right)\exp\;\left(-x^{2}/\left(2\sigma^{2}\right)\right)}\text{.} \end{array}$$

In Definition 5, the variance of this distribution is *σ*^2^. We write Gauss (*σ*^2^) to denote the Gaussian distribution with mean zero and variance *σ*^2^.

**Theorem 2 (Gaussian mechanism [38, 39]).**

Let *f* be a function with *ℓ*_2_-sensitivity and let *ε* ∈ (0,1) be arbitrary. For $\sigma \geq \Delta_{2}f\cdot \sqrt{2\;\ln\;\left(1.25/\delta \right)}/\varepsilon$, the Gaussian mechanism, which adds independently drawn random noise distributed as *Gauss*(*σ*^2^) into each of the *d* components of the output, ensures (*ε*, *δ*)-differential privacy.

In Theorem 2, to ensure (*ε*, *δ*)-differential privacy, we can inject the Gaussian noise with *σ*^2^=2 ln (1.25/*δ*) · (Δ_2_*f*/*ε*)^2^ into the input data directly.

### 2.4. Injecting Noise into the Input Data via Lifting Haar Wavelet

#### 2.4.1. Lifting Scheme of Haar Wavelet

**Figure 1:**
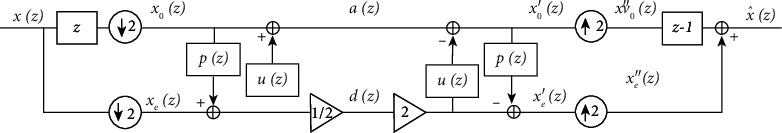
Lifting scheme of Haar wavelet transform.

The lifting scheme of Haar wavelet transform is shown in Figure 1. In Figure 1, *x*(*z*) is the input data, *x*_*o*_ (*z*) and *x*_*e*_ (*z*) denote the odd indexed samples and even indexed samples, respectively. *a* (*z*) and *d* (*z*) are the approximate coefficients and detail coefficients, respectively. For lifting scheme of Haar wavelet, we have *p* (*z*) = −1 and *u* (*z*) = 1/2.

In Figure 1, we have

(6) $$\begin{array}{c} x\left(z\right)=\overset{n-1}{\underset{i=0}{\sum }}x_{i}\cdot z^{-i}\\ =\sum x_{2k}\cdot {\left(z^{2}\right)}^{-k}+z^{-1}\cdot \sum x_{2k+1}\cdot {\left(z^{2}\right)}^{-k}\\ =x_{e}\left(z^{2}\right)+z^{-1}x_{o}\left(z^{2}\right)\text{.} \end{array}$$

Therefore, the approximate coefficients *a* (*z*) and detail coefficients *d* (*z*) can be given as follows:

(7) $$\begin{array}{c} a\left(z\right)=\frac{1}{2}\left(x_{e}\left(z\right)+x_{o}\left(z\right)\right)\text{,} \end{array}$$

(8) $$\begin{array}{c} d\left(z\right)=\frac{1}{2}\left(x_{e}\left(z\right)-x_{o}\left(z\right)\right)\text{.} \end{array}$$

In Figure 1, the lifting structure has the reconstruction property, that is

(9) $$\begin{array}{c} x_{o}^{\prime }\left(z\right)=x_{o}\left(z\right),x_{e}^{\prime }\left(z\right)=x_{e}\left(z\right),\hat{x}\left(z\right)=x\left(z\right)\text{.} \end{array}$$

**Figure 2:**
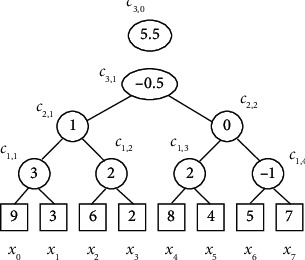
Multilevel lifting Haar wavelet transform.

Figure 1 shows one-level decomposition and reconstruction via lifting Haar wavelet transform. The wavelet transform usually consists of many decomposition levels. We can apply the same procedure to the approximate coefficients *a* (*z*) to get the multilevel Haar wavelet decomposition, as shown in Figure 2.

In Figure 2, the top decomposition level is 3 (*l* = 3), *c*_3,0_ is the approximate coefficient, *c*_*k*,*i*_ (*i* ≠ 0) denotes the *i*th wavelet coefficient in *k*th decomposition level, and *x*_*m*_(*m* ∈ [0,7]) denotes the input data. In Figure 2, we observe that the number of wavelet coefficients in *k*th decomposition level is 2^*l*−*k*^.

In Figure 2, given the Haar wavelet coefficients, any entry *x*_*m*_ can be easily reconstructed as follows:

(10) $$\begin{array}{c} x_{m}=c_{l,0}+\underset{c_{k,i}\in C\backslash \left\{c_{l,0}\right\}}{\sum }\left(c_{k,i}\cdot g_{k,i}\right)\text{,} \end{array}$$

where *c*_*l*,0_ is the approximate coefficient, *c*_*k*,*i*_(*i* ≠ 0) denotes the *i*th wavelet coefficient in *k*th decomposition level, and *g*_*k*,*i*_ equals 1 (−1) if *x*_*m*_ is in the left (right) subtree of *c*_*k*,*i*_, equals 0 if *x*_*m*_ is not in any subtree of *c*_*k*,*i*_. For example,

(11) $$\begin{array}{c} x_{1}=3=c_{3,0}+c_{3,1}+c_{2,1}-c_{1,1}\text{.} \end{array}$$

In Figure 2, if we inject the noise into the approximate coefficients and detail coefficients, then we can obtain the reconstruction data with noise.

#### 2.4.2. Injecting Noise into Haar Wavelet Coefficients

For the noise injected into Haar wavelet coefficients, the tree structure of noise can be obtained by changing the symbol “*c*” and “*x*” to *n* in Figure 2 because they use the same decomposition of multilevel lifting Haar wavelet transform.

Referring to equation (10), the noise *n*_*m*_ that injected into data *x*_*m*_ can be given as follows:

(12) $$\begin{array}{c} n_{m}=n_{l,0}+\underset{n_{k,i}\in N\backslash \left\{n_{l,0}\right\}}{\sum }\left(n_{k,i}\cdot g_{k,i}\right)\text{,} \end{array}$$

where *n*_*l*,0_ is the noise injected into approximate coefficient, *n*_*k*,*i*_ (*i* ≠ 0) denotes the noise injected into the *i*th wavelet coefficient in *k*th decomposition level, and *g*_*k*,*i*_ equals 1 (−1) if *n*_*m*_ is in the left (right) subtree of *n*_*k*,*i*_ and equals 0 if *n*_*m*_ is not in any subtree of *n*_*k*,*i*_.

The range sum of these noise has a special property; that is, some subnoise items can be eliminated when computing some sum of range count. For example, referring to Figure 2 and equation (12), we have

(13) $$\begin{array}{c} \overset{2^{l}-1}{\underset{i=0}{\sum }}n_{i}=\overset{7}{\underset{i=0}{\sum }}n_{i}=8\cdot n_{3,0}\text{.} \end{array}$$

In the above equation, the other subnoise items except *n*_3,0_ have been eliminated. This gives us the inspiration to apply this property to range query for differential privacy.

#### 2.4.3. Getting Input Data with Noise

**Figure 3:**
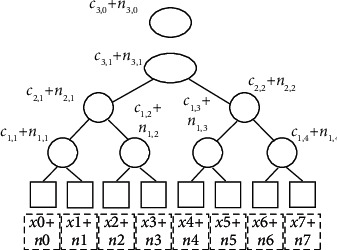
Getting input data with noise.

Based on the above two sections, we reconstruct the input data with noise by using the multilevel lifting Haar wavelet transform. Considering Figure 2, the input data with noise is shown in Figure 3.

In Figure 3, *x*_*m*_(*m* ∈ [0,7]) denotes the input data reconstructed, *n*_*m*_ is the noise injected into data *x*_*m*_. *x*_*m*_+*n*_*m*_ denotes the input data with noise. Referring to equations (10) and (12), we have

(14) $$\begin{array}{c} x_{m}+n_{m}=c_{l,0}+\underset{c_{k,i}\in C\backslash \left\{c_{l,0}\right\}}{\sum }\left(c_{k,i}\cdot g_{k,i}\right)+n_{l,0}+\underset{n_{k,i}\in N\backslash \left\{n_{l,0}\right\}}{\sum }\left(n_{k,i}\cdot g_{k,i}\right)\\ =\left(c_{l,0}+n_{l,0}\right)+\underset{\begin{array}{c} c_{k,i}\in C\backslash \left\{c_{l,0}\right\}\\ n_{k,i}\in N\backslash \left\{n_{l,0}\right\} \end{array}}{\sum }\left(\left(c_{k,i}+n_{k,i}\right)\cdot g_{k,i}\right)\text{,} \end{array}$$

where the meanings of the symbols *c*_*l*,0_, *n*_*l*,0_, *c*_*k*,*i*_, *n*_*k*,*i*_, and *g*_*k*,*i*_ are as stated before.

Based on the analysis of above, we conclude that the input data with noised can be obtained by injecting the noise, such as Laplace noise or Gaussian noise, into the approximate and detail coefficients. Moreover, the noise injected into each input data is the sum of the noise injected into approximate and detail coefficients.

#### 2.4.4. Injecting Noise via Haar Wavelet and Laplace Mechanism

In equation (12), we set *n*_*k*,*i*_ as the noise with the Laplace distribution, as given in Definition 3. We have

(15) $$\begin{array}{c} n_{k,i}∼Lap\left(\frac{\lambda }{2^{k}}\right)\text{,} \end{array}$$

where *λ* is the scale parameter of Laplace distribution and *k* denotes the *k*th decomposition level of lifting Haar wavelet transform.

According to equations (12) and (15), there is

(16) $$\begin{array}{c} n_{m}∼Lap\left(\frac{\lambda }{2^{l}}\right)+\underset{n_{k,i}\in N\backslash \left\{n_{l,0}\right\}}{\sum }\left(Lap\left(\frac{\lambda }{2^{k}}\right)\cdot g_{k,i}\right)\text{,} \end{array}$$

where the symbols of *n*_*m*_, *n*_*k*,*i*_, *n*_*l*,0_, and *g*_*k*,*i*_ are the same as those in equation (12).

Using equations (12) and (16), we can describe the Laplace noise injected into Haar wavelet coefficients, as listed in Table 1.

According to Table 1, letting the range of the range query is *n*_0_ to *n*_7_, we have

(17) $$\begin{array}{c} \overset{2^{l}-1}{\underset{i=0}{\sum }}n_{i}=\overset{7}{\underset{i=0}{\sum }}n_{i}=8\cdot n_{3,0}∼Lap\left(8\cdot \frac{\lambda }{2^{3}}\right)=Lap\left(\lambda \right)\text{.} \end{array}$$

That means the sum of all noise injected into the input data is a noise with Laplace distribution with mean zero and scale *λ*.

Letting *λ*=Δ_1_*f*/*ε*, then *ε*=Δ_1_*f*/*λ*, according to Theorem 1, we conclude the (*ε*, 0)-differential privacy is preserved for the range query from *n*_0_ to *n*_7_ using Laplace mechanism.

According to Table 1, letting the range of the range query is *n*_1_ to *n*_3_, we have

(18) $$\begin{array}{c} n_{1}+n_{2}+n_{3}=3\cdot n_{3,0}+3\cdot n_{3,1}-n_{2,1}-n_{1,1}\text{.} \end{array}$$

Therefore,

(19) $$\begin{array}{c} n_{1}+n_{2}+n_{3}∼Lap\left(3\cdot \frac{\lambda }{2^{3}}\right)+Lap\left(3\cdot \frac{\lambda }{2^{3}}\right)-Lap\left(\frac{\lambda }{2^{2}}\right)-Lap\left(\frac{\lambda }{2^{1}}\right)\text{.} \end{array}$$

As we know, the sum of independent random Laplace variables is not a variable of Laplace distribution, so the compositive noise of range query of *n*_1_+*n*_2_+*n*_3_ that injected into input data *x*_1_+*x*_2_+*x*_3_ is not a noise with Laplace distribution. Therefore, we conclude that it is difficult to judge the level of differential privacy protection based on the Haar wavelet transform and Laplace mechanism.

To solve this problem, we consider adopting the Gaussian mechanism for the differential privacy via Haar wavelet transform in the next section.

## 3. Injecting Noise into Haar Wavelet Coefficients via Gaussian Mechanism

To inject Gaussian noise into Haar wavelet coefficients in Figure 3, we can set *n*_*k*,*i*_ as the noise with the Gaussian distribution, as given in Definition 5.

Let

(20) $$\begin{array}{c} n_{k,i}∼Gauss\left(\frac{3\sigma^{2}}{4^{k}}\right)\text{,} \end{array}$$

where 3*σ*^2^/4^k^ is the variance of Gaussian distribution.

According to equations (12) and (20), we have

(21) $$\begin{array}{c} n_{m}∼Gauss\left(\frac{3\sigma^{2}}{4^{l}}\right)+\underset{n_{k,i}\in N\backslash \left\{n_{l,0}\right\}}{\sum }\left(Gauss\left(\frac{3\sigma^{2}}{4^{k}}\right)\cdot g_{k,i}\right)\text{,} \end{array}$$

where the symbols of *n*_*m*_, *n*_*k*,*i*_, *n*_*l*,0_, and *g*_*k*,*i*_ are same as those in equation (12).

Using equations (20) and (21), we can describe the Gaussian noise injected into Haar wavelet coefficients, as listed in Table 2.

**Theorem 3 .**

Suppose that *X*_1_ and *X*_2_ are independent random variables, and *X*_*i*_ has Gaussian distribution with mean zero and variance *σ*_*i*_^2^ for *i* ∈ {1,2}. Then, *X*_1_ ± *X*_2_ is Gaussian distribution with mean zero and variance *σ*_1_^2^+*σ*_2_^2^; *kX*_1_ is Gaussian distribution with mean zero and variance (*kσ*_1_)^2^.

The proof of Theorem 3 will not be given because it is a basic property of Gaussian distribution.

According to Table 2 and Theorem 3, there is

(22) $$\begin{array}{c} \overset{2^{l}-1}{\underset{i=0}{\sum }}n_{i}=\overset{7}{\underset{i=0}{\sum }}n_{i}=8\cdot n_{3,0}=Gauss\left(8^{2}\cdot \frac{3\sigma^{2}}{4^{3}}\right)=Gauss\left(3\sigma^{2}\right)\text{.} \end{array}$$

That means the sum of all noise injected into the input data is a noise with Gaussian distribution. We analyze the distribution of the noise injected into each input data as follows.

**Theorem 4 .**

Injecting Gaussian noise with variance *σ*_*k*_^2^=3*σ*^2^/4^*k*^ into the Haar wavelet coefficients in the *k*th decomposition level (the maximum decomposition level is *l*, as shown in Figure 3), the noise injected into each input data via Haar wavelet reconstruction is Gaussian noise with variance (1 + 2/4^*l*^) *σ*^2^.

**Proof**

According to Definition 5, Theorem 3, Table 2, and equation (21), we have

(23) $$\begin{array}{c} n_{m}∼Gauss\left(\frac{3\sigma^{2}}{4^{l}}\right)\pm \overset{1}{\underset{i=l}{\sum }}Gauss\left(\frac{3\sigma^{2}}{4^{i}}\right)\text{,}\\ ∼Gauss\left(\left(\frac{1}{4^{l}}+\overset{l}{\underset{i=1}{\sum }}\left(1/4^{i}\right)\right)\cdot 3\sigma^{2}\right)\text{,}\\ ∼Gauss\left(\left(1+\frac{2}{4^{l}}\right)\cdot \sigma^{2}\right)\text{.} \end{array}$$

The proof is completed.

Using Theorems 2 and 4, we can obtain the (*ε*, *δ*)-differential privacy with variance (1 + 2/4^*l*^) *σ*^2^ for each input data under the framework of Gaussian mechanism via Haar wavelet transform.

**Theorem 5 (Differential privacy using Gaussian mechanism and Haar wavelet).**

Let *f* be a function with *ℓ*_2_-sensitivity, let *ε* ∈ (0,1) be arbitrary, and let $\sigma =\Delta_{2}f\cdot \sqrt{2\;\ln\;\left(1.25/\delta \right)}/\varepsilon$. The mechanism adopting Gaussian and Haar wavelet, which adds Gaussian noise with variance *σ*_*k*_^2^=3*σ*^2^/4^*k*^ into the Haar wavelet coefficients in the *k*th decomposition level (Figure 3), ensures (*ε*, *δ*)-differential privacy.

**Proof**

In Theorem 4, if the Gaussian noise with variance *σ*_*k*_^2^=3*σ*^2^/4^*k*^is injected into the wavelet coefficients, the reconstructed data will be the one with the Gaussian noise with variance $\sqrt{1+2/4^{l}}\sigma$. According to Theorem 2, the condition of $\sqrt{1+2/4^{l}}\sigma \geq \Delta_{2}f\cdot \sqrt{2\;\ln\;\left(1.25/\delta \right)}/\varepsilon$ should be satisfied. Therefore, letting $\sigma =\Delta_{2}f\cdot \sqrt{2\;\ln\;\left(1.25/\delta \right)}/\varepsilon$, the condition is met and the proof is completed.

We simulate the process of injecting Gaussian noise with = 12 into wavelet coefficients with 15-level decomposition using Theorem 4 and injecting Gaussian noise into input data directly and draw the noise-count figures as follows.

**Figure 4:**
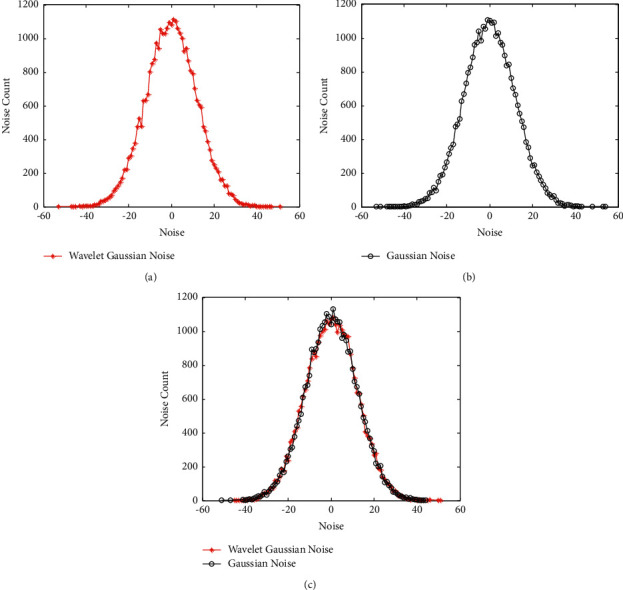
Comparison between injecting Gaussian noise into wavelet coefficients using Theorem 4 and injecting Gaussian noise into input data directly.

Figure 4(a) shows the count of the noise injected into input data by injecting the Gaussian noise with *σ* = 12 into Haar wavelet coefficients with 15-level decomposition using equation (20); Figure 4(b) denotes the noise count by injecting Gaussian noise with mean zero and variance (1 + 2/4^*l*^) *σ*^2^ into the input data directly. In Figure 4(c), we draw the two curves together and we find that they are almost overlapped. Figure 4(c) shows that the method injecting Gaussian noise into Haar wavelet coefficients has the same level of differential privacy protection as the method injecting Gaussian noise into the input data directly. In this study, we will focus on the application of range query.

According to Theorems 3−5, we find that the distribution of noise for the range query using Gaussian mechanism and Haar wavelet is a Gaussian distribution. Therefore, we can calculate the variance of noise easily, for example, as listed in Table 2, we have

(24) $$\begin{array}{c} n_{1}+n_{2}+n_{3}=n_{3,0}+n_{3,1}+n_{2,1}-n_{1,1}\\ +n_{3,0}+n_{3,1}-n_{2,1}+n_{1,2}\\ +n_{3,0}+n_{3,1}-n_{2,1}-n_{1,2}\\ =3n_{3,0}+3n_{3,1}-n_{2,1}-n_{1,1}\text{,}\\ ∼3Gauss\left(\frac{3\sigma^{2}}{4^{3}}\right)+3Gauss\left(\frac{4\sigma^{2}}{4^{3}}\right)-Gauss\left(\frac{3\sigma^{2}}{4^{2}}\right)-Gauss\left(\frac{3\sigma^{2}}{4^{1}}\right)\text{,}\\ ∼Gauss\left(3^{2}\cdot \frac{3\sigma^{2}}{4^{3}}+3^{2}\cdot \frac{3\sigma^{2}}{4^{3}}+\frac{3\sigma^{2}}{4^{2}}+\frac{3\sigma^{2}}{4^{1}}\right)\text{,}\\ ∼Gauss\left(\frac{57}{32}\sigma^{2}\right)\text{.} \end{array}$$

From this example, we find that some noises (such as *n*_2,1_ and −*n*_2,1_, *n*_1,2_ and −*n*_1,2_) are eliminated by the operation of addition. According to Theorem 4, the variance of noise injected into each input data should be (1+2/4^3^)*σ*^2^ for *l* = 3. The total noise variance is 3*∗*(1+2/4^3^)*σ*^2^=(99/32)*∗σ*^2^. Compared with equation (24), we conclude that, for the range query, the noise actually added into a certain range of the original data is much less than the sum of the noise at each data. Therefore, it is a very important property for Gaussian mechanism to be used on range query.

## 4. Noise of Range Query under the Framework of Haar Wavelet and Gaussian Mechanism

In this section, we discuss how to compute the noise of the range query under the framework of Haar wavelet transform and Gaussian mechanism. First, we give the computing method for the variance of range query in detail. Second, the interval of the range query when obtaining the maximum variance is introduced. Third, to speed up the computing of maximum variance, we observe the results of the range-count interval when getting the maximum variance and give a speed computing method. Finally, we give a coarse estimation of the maximum variance of range query as a function expression.

### 4.1. Computing Method for the Variance of Range Query

In Figure 3, we choose the Gaussian noise and inject them into the approximate coefficient and each wavelet coefficient. The variance of Gaussian noise injected into approximate coefficient is 3*σ*^2^/4^3^. The variance of Gaussian noise injected into each wavelet coefficient is 3*σ*^2^/4^*k*^ for level *k*(*k* ∈ [1,3]). The relationship between decomposition level and variance of noise is listed in Table 3.

The noise-sum of range query via Haar wavelet transform for interval *S* can be given by the following equation (Figure 3):

(25) $$\begin{array}{c} n_{sum}=\left|S\right|\cdot n_{l,0}+\underset{n_{k,i}\in N\backslash \left\{n_{l,0}\right\}}{\sum }\left(n_{k,i}\cdot \left(\alpha \left(n_{k,i}\right)-\beta \left(n_{k,i}\right)\right)\right)\text{,} \end{array}$$

where *S* is the interval of any range query, *n*_*k*,*i*_ presents the *i*th noise injected into wavelet coefficient in *k*th decomposition level, *α*(*n*_*k*,*i*_) denotes the number of left leaves in the left subtree of *n*_*k*,*i*_ that are contained in *S*, and *β*(*n*_*k*,*i*_) denotes the number of right leaves in the right subtree of *n*_*k*,*i*_ that are contained in *S* (Figure 3).

Now we analyze the noise variance of range query. According to equation (20), we know that the noise injected into approximate coefficient is *n*_*l*,0_ and its variance is 3*σ*^2^/4^*l*^; the noise injected into wavelet coefficient is *n*_*k*,*i*_ and its variance is 3*σ*^2^/4^*k*^. Therefore, according to Theorem 3, we can compute the noise-variance of range query by replacing *n*_*l*,0_ and *n*_*k*,*i*_ with 3*σ*^2^/4^*l*^ and 3*σ*^2^/4^*k*^ in equation (25), respectively.

(26) $$\begin{array}{c} \sigma_{sum}^{2}={\left|S\right|}^{2}\cdot \frac{3\sigma^{2}}{4^{l}}+\underset{n_{k,i}\in N\backslash \left\{n_{l,0}\right\}}{\sum }\left(\frac{3\sigma^{2}}{4^{k}}{\left(\alpha \left(n_{k,i}\right)-\beta \left(n_{k,i}\right)\right)}^{2}\right)\\ =\left({\left|S\right|}^{2}\cdot \frac{1}{4^{l}}+\underset{n_{k,i}\in N\backslash \left\{n_{l,0}\right\}}{\sum }\left(\frac{1}{4^{k}}{\left(\alpha \left(n_{k,i}\right)-\beta \left(n_{k,i}\right)\right)}^{2}\right)\right)\cdot 3\sigma^{2}\text{.} \end{array}$$

To compute the value of *σ*_*sum*_^2^, we need to calculate the values of *α*(*n*_*k*,*i*_) and *β*(*n*_*k*,*i*_) firstly. In Figure 3, the length of interval of leaves in the subtree of *n*_*k*,*i*_ is 2^*k*^. The left point of this interval has the subscript (*i* − 1) · 2^*k*^ and the right point of this interval has the subscript *i* · 2^*k*^ − 1. Therefore, the subtree of *n*_*k*,*i*_ has the subscript interval of leaves.

(27) $$\begin{array}{c} S_{n_{k,i}}=\left[\left(i-1\right)\cdot 2^{k},i\cdot 2^{k}-1\right]\text{.} \end{array}$$

For example, the wavelet coefficient *n*_2,2_ in Figure 3 has the subscript interval of leaves [4, 7].

According to equation (27), we can obtain the left-half interval [*α*_*L*_, *α*_*R*_]and right-half interval [*β*_*L*_, *β*_*R*_] of *S*_*n*_*k*,*i*_:

(28) $$\begin{array}{c} \left[\alpha_{L},\alpha_{R}\right]=\left[\left(i-1\right)\cdot 2^{k},i\cdot 2^{k}-2^{k-1}-1\right]\text{,}\\ \left[\beta_{L},\beta_{R}\right]=\left[i\cdot 2^{k}-2^{k-1},i\cdot 2^{k}-1\right]\text{.} \end{array}$$

Therefore, *α*(*n*_*k*,*i*_) and *β*(*n*_*k*,*i*_) can be given by computing the number of intersection between *S* and [*α*_*L*_, *α*_*R*_], *S* and [*β*_*L*_, *β*_*R*_], respectively.

(29) $$\begin{array}{c} \alpha \left(n_{k,i}\right)=\left|S\cap \left[\alpha_{L},\alpha_{R}\right]\right|\text{,}\\ \beta \left(n_{k,i}\right)=\left|S\cap \left[\beta_{L},\beta_{R}\right]\right|\text{.} \end{array}$$

Let *S*=[*S*_*L*_, *S*_*R*_], where *S*_*L*_ and *S*_*R*_ denote the left and right points of the given range query interval, respectively. Therefore, we have

(30) $$\begin{array}{c} \alpha \left(n_{k,i}\right)=\left|\left[S_{L},S_{R}\right]\cap \left[\alpha_{L},\alpha_{R}\right]\right|\text{,} \end{array}$$

(31) $$\begin{array}{c} \beta \left(n_{k,i}\right)=\left|\left[S_{L},S_{R}\right]\cap \left[\beta_{L},\beta_{R}\right]\right|\text{,} \end{array}$$

where *k* ∈ [1, *l*] and *i* ∈ [1, 2^*l*−*k*^] (Figure 3).

### 4.2. Maximum Variance of Range Query

The aim of this study is to obtain the maximum value of range query for any fixed maximum decomposition level *l* (the number of input data is 2^*l*^). According to equation (26), we have

(32) $$\begin{array}{c} \sigma_{sumMax}^{2}=\max\;\left({\left|S\right|}^{2}\cdot \frac{1}{4^{l}}+\underset{n_{k,i}\in N\backslash \left\{n_{l,0}\right\}}{\sum }\left(\frac{1}{4^{k}}{\left(\alpha \left(n_{k,i}\right)-\beta \left(n_{k,i}\right)\right)}^{2}\right)\right)\cdot 3\sigma^{2}\text{.} \end{array}$$

In equation (32), the given parameter is *l*. To compute the value of *σ*_*sumMax*_^2^, we need to calculate any range query interval *S*=[*S*_*L*_, *S*_*R*_] in all data and obtain the count *α*(*c*_*k*,*i*_) and *β*(*c*_*k*,*i*_) using equations (30) and (31). We give the pseudocode of computing *σ*_*sumMax*_^2^ as follows:

Algorithm 1 illustrates the details of the algorithm of computing *σ*^2^_sumMax_ for fix *l* (*l* ≥ 2). Step 1 is the initialization of *σ*^2^_sumMax_. Steps 2 to 3 are the range loop of *S*_*L*_ and *S*_*R*_. Step 5 is the loop of the subscript of decomposition level *k*. Step 6 is the loop of the subscript of the wavelet coefficient in *k*th decomposition level. Step 7 is the computation of the *α*_*L*_, *α*_*R*_, *β*_*L*_, and *β*_*R*_ of *n*_*k*, *i*_. Steps 8 to 13 denote the computation of *α* (*n*_*k*, *i*_). Steps 14 to 19 denote the computation of *β* (*n*_*k*, *i*_). Step 20 is the computation of right part of *σ*^2^_sum_ using equation (26). Step 23 is the computation of *σ*^2^_sum_ using equation (26). Steps 24 to 26 denote the computation of *σ*^2^_sumMax_ using equation (34). Step 29 denotes the output of Algorithm 1.

According to Algorithm 1, we calculate the values of *σ*^2^_sumMax_, *S*_*L*_, and *S*_*R*_, as listed in Table 4.

In Table 4, *S*_*R*_ − *S*_*L*_ + 1 denotes the length of interval for the *σ*^2^_sumMax_. It will take a very long time to compute the *σ*^2^_sumMax_ using Algorithm 1 when *l* > 14, so we need to find some method to speed up Algorithm 1.

### 4.3. Speeding Algorithm for Computing the Maximum Variance of Range Query

Observing the values of *S*_*L*_ and *S*_*R*_ in Table 4, we give some statistical rules to compute them directly in Table 5.

According to Table 5, we can compute *σ*^2^_sumMax_ using the following algorithm:

Algorithm 2 illustrates the details of the algorithm of computing *σ*^2^_sumMax,*l*_ for any *l* (*l* ≥ 2). Step 1 is the initialization of *S*_*L*_ and *S*_*R*_. Step 2 is the loop of the maximum decomposition level *l*. Steps 3 to 7 denote the computation of *S*_*L*_ and *S*_*R*_ according to the maximum decomposition level *l*. Step 10 is the loop of the subscript of decomposition level *k*. Step 11 is the loop of the subscript of the wavelet coefficient in *k*th decomposition level. Step 12 is the computation of the *α*_*L*_, *α*_*R*_, *β*_*L*_, and *β*_*R*_ of *n*_*k*, *i*_. Steps 13 to 18 denote the computation of *α* (*n*_*k*, *i*_). Steps 19 to 24 denote the computation of *β* (*n*_*k*, *i*_). Step 25 is the computation of right part of *σ*^2^_sum_ using equation (26). Step 28 is the computation of *σ*^2^_sum_ using equation (26). Step 30 denotes the output of Algorithm 2 for any *l*.

*S*_*L*_ and *S*_*R*_ can also be given directly by simplifying the results in Table 5.

If *l* is an even number,

(33) $$\begin{array}{c} S_{L}=\frac{2^{l-2}-1}{3},S_{R}=\frac{11\times 2^{l-2}-2}{3}\text{.} \end{array}$$

If *l* is an odd number,

(34) $$\begin{array}{c} S_{L}=\frac{2^{l-2}+1}{3},S_{R}=\frac{11\times 2^{l-2}-4}{3}\text{.} \end{array}$$

Therefore, we give the values of *σ*^2^_sumMax_, *S*_*L*_, *S*_*R*_, and *S*_*R*_ − *S*_*L*_ + 1 for *l* from 2 to 30 in Table 6.

In Table 6, *S*_*L*_ and *S*_*R*_ denote the left and right points of the range query interval when the *σ*^2^_sumMax_ is met. *S*_*R*_ − *S*_*L*_ + 1 denotes the length of interval for the *σ*^2^_sumMax_. In Table 6, we observe that *σ*^2^_sumMax_ will increase about (2/3) *σ*^2^ if the parameter *l* increases 1.

### 4.4. Coarse Estimation of the Maximum Variance of Range Query

In previous sections, the maximum variance of range queries via Gaussian mechanism and Haar wavelet transform is given for any *l*. But it is obtained using a computer program, not from a function expression. In this section, the coarse estimation of the maximum variance is given in Theorem 6, and it is a function expression with parameters *l* and *σ*^2^.

**Theorem 6 (Coarse estimation of the maximum variance).**

Let *N* be a set of independent Gaussian noise *n*_*k*,*i*_ ∈ *N* with a variance 3*σ*^2^/4^*k*^, which is injected into one-dimensional Haar wavelet coefficients and approximate coefficient (Figure 3). Suppose *l*=log_2_|*N*|, that means the number of Gaussian noise injected into Haar wavelet coefficients and approximate coefficient is 2^*l*^ (the number of input data is also 2^*l*^). Let *M* be the noisy data reconstructed from *C* + *N* (*C* is the set of one-dimensional Haar wavelet coefficients of the input data, refer to Figure 2). Then, for any range query answered using *M*, the variance of noise in the answer is at most ((6*l*+9)/4)*σ*^2^.

**Proof**

Referring to Figure 3 and equation (26), we observe that for any noise *n*_*k*,*i*_, if none of the leaves under *n*_*k*,*i*_ is contained in *S*, then there is *α*(*n*_*k*,*i*_)=*β*(*n*_*k*,*i*_)=0. On the other hand, if all leaves under *n*_*k*,*i*_ are covered by *S*, then *α*(*n*_*k*,*i*_)=*β*(*n*_*k*,*i*_)=2^*k*−1^. Therefore, *α*(*n*_*k*,*i*_) − *β*(*n*_*k*,*i*_) ≠ 0, if and only if the left or right subtree of *n*_*k*,*i*_ partially intersects *S*. At any level of the decomposition tree except for the *l*th level, there exist at most two such noises. At the level *l*, at most one such noise that letting the condition *α*(*n*_*k*,*i*_) − *β*(*n*_*k*,*i*_) ≠ 0 be sufficient.

Considering a noise *n*_*k*,*i*_ at level *k* (*k* ∈ [1, *l*]), such that *α*(*n*_*k*,*i*_) − *β*(*n*_*k*,*i*_) ≠ 0. Since the left (right) subtree of *n*_*k*,*i*_ contains at most 2^*k*−1^ leaves, we have *α*(*n*_*k*,*i*_), *β*(*n*_*k*,*i*_) ∈ [0, 2^*k*−1^]. So, there is |*α*(*n*_*k*,*i*_) − *β*(*n*_*k*,*i*_)| ≤ 2^*k*−1^. Therefore, the variance of the range query about the noise *n*_*k*,*i*_(*k* ∈ [1, *l*]) at most is

(35) $$\begin{array}{c} {\left(\alpha \left(n_{k,i}\right)-\beta \left(n_{k,i}\right)\right)}^{2}\cdot \left(\frac{3\sigma^{2}}{4^{k}}\right)\leq 4^{k-1}\cdot \left(\frac{3\sigma^{2}}{4^{k}}\right)=3\sigma^{2}/4\text{.} \end{array}$$

On the other hand, the noise in the approximate coefficient (*n*_*l*,0_) has a variance at most:

(36) $$\begin{array}{c} {\left(2^{l}\right)}^{2}\cdot \left(\frac{3\sigma^{2}}{4^{l}}\right)=4^{l}\cdot \left(\frac{3\sigma^{2}}{4^{l}}\right)=3\sigma^{2}\text{.} \end{array}$$

Therefore, the total variance injected into wavelet coefficients of 1 to *l* − 1 level is 2 · (*l* − 1) · 3*σ*^2^/4, and the variance injected into wavelet coefficients of level *l* is 1 · 3*σ*^2^/4. According to equation (26), the variance of noise at most is

(37) $$\begin{array}{c} {3\sigma }^{2}+2•\left(l-1\right)\cdot \frac{{3\sigma }^{2}}{4}+1•\frac{{3\sigma }^{2}}{4}=\left(\frac{6l+9}{4}\right)\sigma^{2}, \end{array}$$

which completes the proof.

This conclusion in Theorem 6 can also be obtained by observing Table 2. Now, we give the intuitive explanation of Theorem 6.

According to Table 2, we can give a coarse estimation of the maximum variance of range query. In Table 2, we insert a row at the bottom to calculate the maximum variance sum for each column. The new table is shown as follows.

In Table 7, each value of the last row is given by calculating the maximum sum of variance of some continued parts for each column according to Theorem 3. For example, the value of column 2 in the last row, 3*σ*^2^, is calculated by range from *n*_0_ to *n*_7_:

(38) $$\begin{array}{c} 8n_{3,0}∼8Gauss\left(\frac{3\sigma^{2}}{4^{3}}\right)=Gauss\left(8^{2}\cdot \frac{3\sigma^{2}}{4^{3}}\right)=Gauss\left(3\sigma^{2}\right)\text{.} \end{array}$$

The value of column 3 in the last row, 3*σ*^2^/4, is calculated by range from *n*_0_ to *n*_3_ or from *n*_4_ to *n*_7_:

(39) $$\begin{array}{c} \pm 4n_{3,1}∼4Gauss\left(\frac{3\sigma^{2}}{4^{3}}\right)=Gauss\left(4^{2}\cdot \frac{3\sigma^{2}}{4^{3}}\right)=Gauss\left(\frac{3\sigma^{2}}{4}\right)\text{.} \end{array}$$

The value of column 4 in the last row, 3*σ*^2^/2, is calculated by range from *n*_2_ to *n*_5_:

(40) $$\begin{array}{c} -2n_{2,1}+2n_{2,2}∼2Gauss\left(\frac{3\sigma^{2}}{4^{2}}\right)+2Gauss\left(\frac{3\sigma^{2}}{4^{2}}\right)\text{,}\\ ∼Gauss\left(2^{2}\cdot \frac{3\sigma^{2}}{4^{2}}\right)+Gauss\left(2^{2}\cdot \frac{3\sigma^{2}}{4^{2}}\right)\text{,}\\ ∼Gauss\left(2\cdot 2^{2}\cdot \frac{3\sigma^{2}}{4^{2}}\right)\text{,}\\ ∼Gauss\left(\frac{3\sigma^{2}}{2}\right)\text{.} \end{array}$$

The value of column 5 in the last row, 3*σ*^2^/2, is calculated by range from *n*_1_ to *n*_2_ or from *n*_3_ to *n*_4_ or from *n*_5_ to *n*_6_:

(41) $$\begin{array}{c} \begin{array}{c} -n_{1,1}+n_{1,2}∼Gauss\left(3\sigma^{2}/4+3\sigma^{2}/4\right)=Gauss\left(3\sigma^{2}/2\right)\\ -n_{1,2}+n_{1,3}∼Gauss\left(3\sigma^{2}/4+3\sigma^{2}/4\right)=Gauss\left(3\sigma^{2}/2\right)\\ -n_{1,3}+n_{1,4}∼Gauss\left(3\sigma^{2}/4+3\sigma^{2}/4\right)=Gauss\left(3\sigma^{2}/2\right) \end{array}\text{.} \end{array}$$

Therefore, we can obtain an estimation of the maximum variance of range query as follows:

(42) $$\begin{array}{c} \sigma_{sumMaxEstim}^{2}=3\sigma^{2}+\frac{3}{4}\sigma^{2}+\frac{3}{2}\sigma^{2}+\frac{3}{2}\sigma^{2}=\frac{27}{4}\sigma^{2}=\frac{6*3+9}{4}\sigma^{2}\text{.} \end{array}$$

In Table 7, the wavelet decomposition level is 3 (the number of input data is 2^3^). Supposing that the decomposition level is *l*, then we can give an estimation of the maximum variance of range query:

(43) $$\begin{array}{c} \sigma_{sumMaxEstim}^{2}=\left(\frac{6l+9}{4}\right)\sigma^{2}\text{.} \end{array}$$

Note that equation (43) gives the same result with the conclusion in Theorem 6.

Comparing the estimation maximum variances (equation (43)) with the real maximum variances (Table 6), we find that

(44) $$\begin{array}{c} \sigma_{sumMax}^{2}\ll \sigma_{sumMaxEstim}^{2}\text{.} \end{array}$$

*σ*_*sumMaxEstim*_^2^ provides a function expression using parameters *l* and *σ*^2^ for the maximum variance of range query via Haar wavelet transform, but it has a very large error comparing the real maximum variance, comparing equation (43) with Table 6. Therefore, for the analysis of the practical applications, we prefer to use the maximum variance *σ*^2^_sumMax_, as listed in Table 6.

## 5. Experimental Verification

This section introduces the experimental verification of the proposed framework, that is, the computing of maximum variance for range query based on Gaussian mechanism and lifting Haar wavelet. We use the dataset age, which contains census records of individuals from the United States. The age has 107, 974, and 787 records, each of which corresponds to the age of an individual, extracted from the IPUM's census data of the United States [40]. The ages range from 0 to 135 and just have 128 values (ages 121, 123, 127, 128, 131, 132, 133, and 134 are empty). We count the number of each age and give the histogram of age as the input file of our experiments. Given a query length *L*, we test all the possible range queries with length *L* and report the maximum variance of the range query for input data.

The noise injected into the input data via Gaussian mechanism is Gaussian noise. The variance of Gaussian noise is the sample variance. Therefore, the sample variance is adopted in this study and is computed by the following equation:

(45) $$\begin{array}{c} Var\left(n\right)=\frac{1}{m-1}{\overset{m}{\underset{i=1}{\sum }}\left(n_{i}-\frac{1}{m}\overset{m}{\underset{j=1}{\sum }}n_{j}\right)}^{2}\text{,} \end{array}$$

where *n*_*i*_ (or *n*_*j*_) is the noise injected into the input data and *m* is the number of the input data.

We research the maximum variance of the range query of noise when *ε* chosen in the set {0.5, 0.75, 1.0, 1.25} and *δ* chosen in the set {0.1, 0.01, 0.001}. For each special *ε*, we draw the maximum variance of the range count of noise using Gaussian mechanism and Gaussian mechanism with Haar wavelet transform when *δ* is equal to 0.1, 0.01, and 0.001.

For any *ε* and *δ*, we can calculate the *σ* of Gaussian noise using the equation $\sigma =\sqrt{2\;\ln\;\left(1.25/\delta \right)}/\varepsilon$ in Theorem 5, and the results are given in Table 8.

To compute the variance, we inject the Gaussian noise into the input data or the Haar wavelet coefficients 10000 times. To compute the maximum variance of the range query with fixed *ε* and *δ*, each variance of the range query for range size *k* needs to be computed firstly. Then, the maximum value of variance for range size *k* can be given by comparing all the variance of the *k*-range queries.

**Figure 5:**
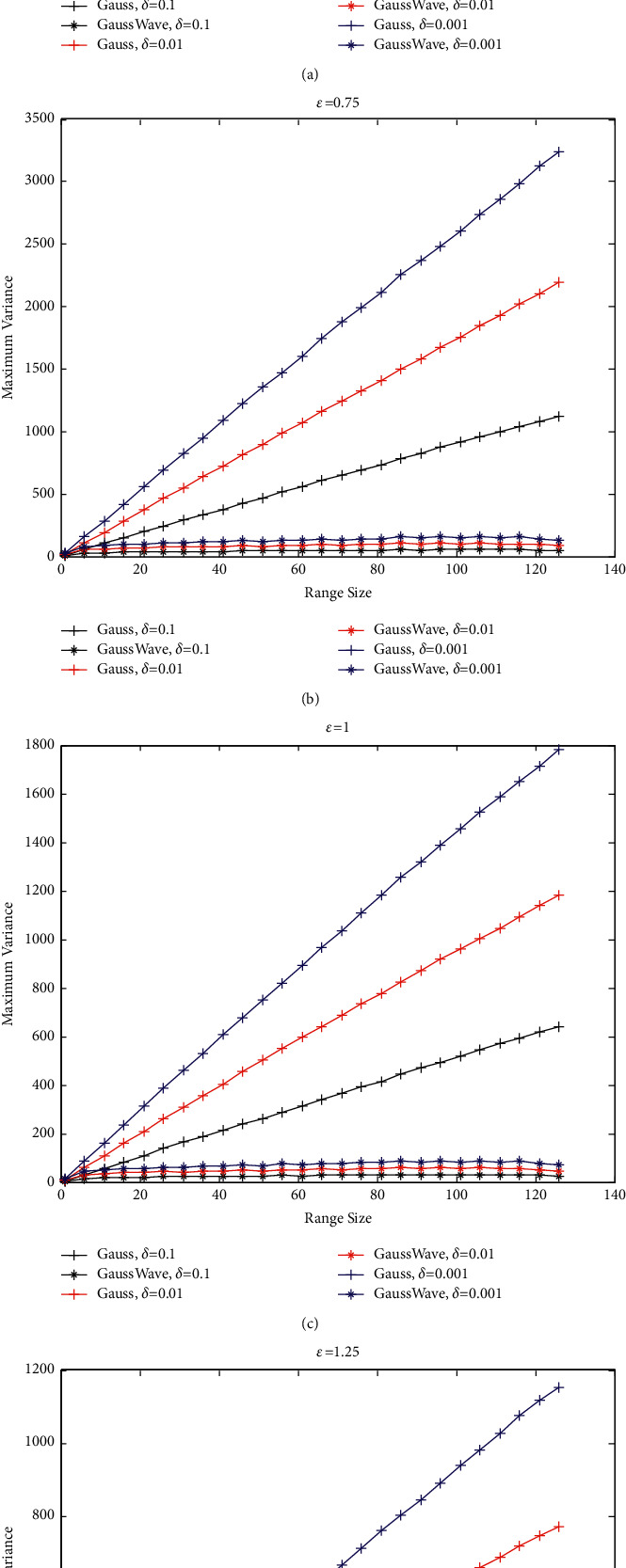
Maximum variance of range query using Gaussian mechanism and Gaussian mechanism with Haar wavelet on age (the United States). (a) *ε* = 0.5. (b) *ε* = 0.75. (c) *ε* = 1.0. (d) *ε* = 1.25.

For input data with length 128 (such as 128 histogram), we can draw the maximum variance diagram of the range query using Gaussian mechanism and Gaussian mechanism with Haar wavelet transform for any range sizes, as shown in Figure 5.

In Figure 5, “Gauss” means injecting noise into each histogram data via Gaussian mechanism directly and then gets the noise of range query by the operation of addition. First, “GaussWave” denotes injecting noise into the lifting Haar wavelet coefficients using Theorem 5. Second, the noise injected into each histogram is obtained by the inverse wavelet transform. Finally, the range query for any range size is obtained by injecting the noise together.

In Figure 5, we observe that the maximum variance is increasing linearly with the “range size” for “Gauss.” In Figure 5, for any *ε* and *δ*, the maximum variance of the noise using “GaussWave” method is far less than the noise using “Gauss” method.

**Figure 6:**
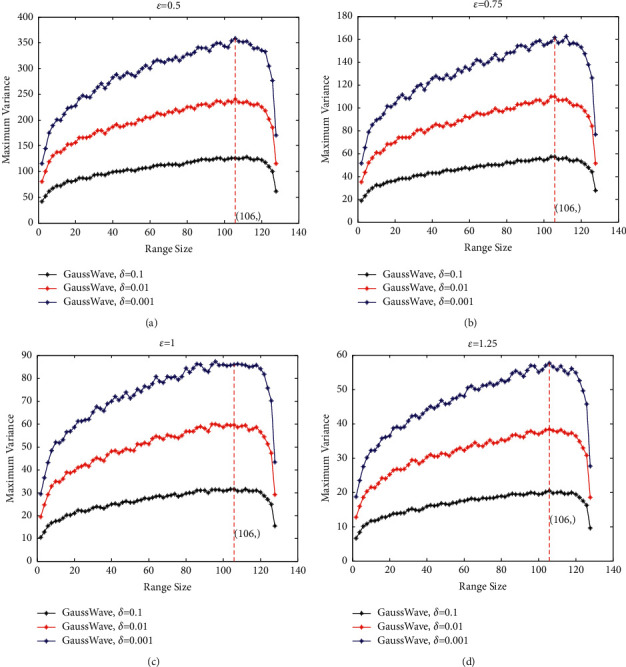
Maximum variance of range query using Gaussian mechanism with Haar wavelet on age (the United States). (a) *ε* = 0.5. (b) *ε* = 0.75. (c) *ε* = 1.0. (d) *ε* = 1.25.

To observe the variation tendency of “GaussWave” in Figure 5, we just draw the maximum variance diagram of the range query using Gaussian mechanism with Haar wavelet transform, as shown in Figure 6.

In Figure 6, for any *ε* and *δ*, the maximum variance of the noise using “GaussWave” method increases with the increase of range size before it gets the maximum value, and it will decrease with the increase of range size after it has gotten the maximum value.

In Table 6, we observe that the max-variance of range query via Gaussian mechanism with Haar wavelet is 6.248291 *∗* *σ*^2^ when *l* = 7 (2^7^ = 128) and the length of interval is 106. Considering the value of *σ* in Table 8, we give the comparison of maximum value between experimental result and theoretical analysis for range query in Table 9.

In Table 9, the column “max-value” presents the experimental result of the maximum value of maximum variance for range query. The column “*σ*^2^_sumMax_” presents the result of theoretical analysis and computed using the formula *σ*^2^_sumMax_ = 6.248291 *∗* *σ*^2^. The last column “difference” is the difference of columns “max-value” and “*σ*^2^_sumMax_.” In Table 9, we find that the results of experiment and theoretical analysis are in substantial agreement.

This section gives the experimental verification of the framework of the maximum variance computing for range query. This framework on privacy preserving is built using Gaussian mechanism and Haar wavelet. In Figure 6, for any *ε* and *δ*, the maximum variance of the noise increases with the increase of range size before it gets the maximum value of 106, and it will decrease with the increase of range size after it has gotten the maximum value. In Table 9, the experimental value and the theoretical value of maximum variance for range count are compared, and the results show that they are in substantial agreement.

## 6. Conclusions

In this study, we proposed a new differential privacy framework via Haar wavelet transform and Gaussian mechanism for the range query. The theorems for how to inject Gaussian noise into the Haar wavelet coefficients are given. The noise of range query under the theoretical framework of Haar wavelet and Gaussian mechanism is analyzed. The algorithm to compute the maximum variance of any range query for any given parameter *l* is introduced. A coarse estimation of the maximum variance of range query using a function expression is given. The experimental results show that the maximum variance of the noise using Gaussian mechanism and Haar wavelet is far less than the noise using Gaussian mechanism. The experimental verification of the computing of maximum variance for range query based on lifting Haar wavelet and Gaussian mechanism is proposed, and the results show the experimental value and the theoretical value of maximum variance for range count are substantial agreement. For future work, we plan to apply our method to the privacy protection of histogram publication. Furthermore, we want to investigate how to assemble our method and machine learning algorithm, such as the decision tree and random forest.

## Figures and Tables

**Algorithm 1:**
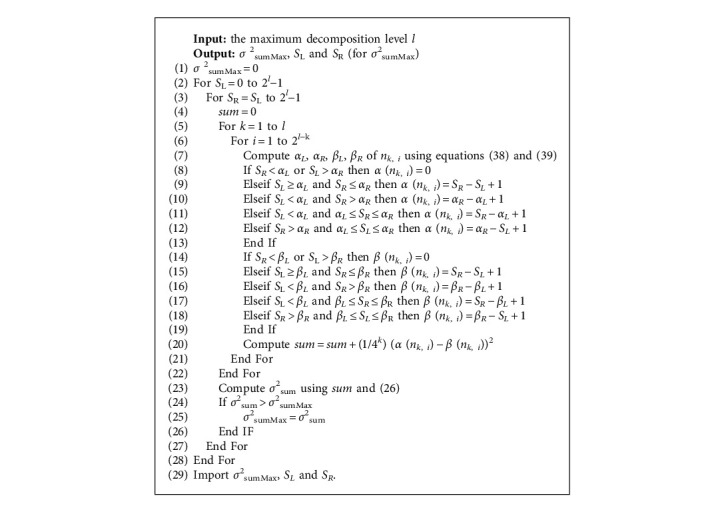
Compute *σ*^2^_sumMax_for fix *l* (*l* ≥ 2).

**Algorithm 2:**
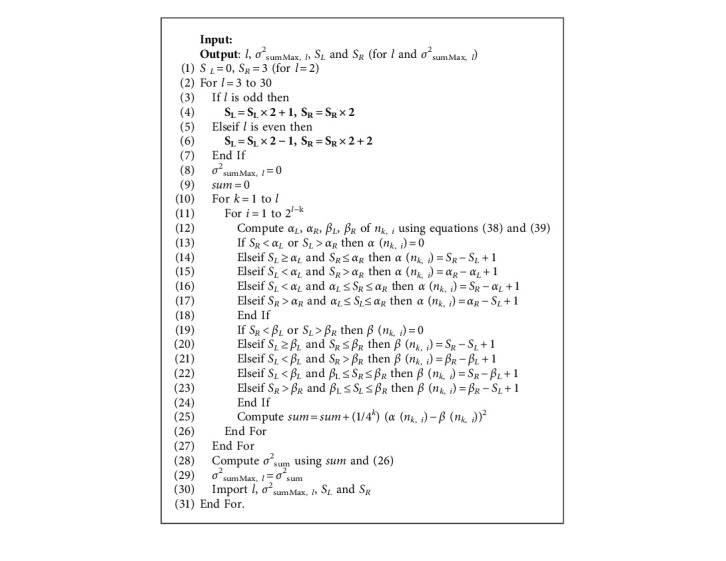
Compute *σ*^2^_sumMax, *l*_ for any *l* (*l* ≥ 2).

**Table 1:** Laplace noise injected into Haar wavelet coefficients.

*n *_0_=	*n *_3, 0_	+*n*_3, 1_	+*n*_2, 1_	+*n*_1, 1_
*n *_1_=	*n *_3, 0_	+*n*_3, 1_	+*n*_2, 1_	−*n*_1, 1_
*n *_2_=	*n *_3, 0_	+*n*_3, 1_	−*n*_2, 1_	+*n*_1, 2_
*n *_3_=	*n *_3, 0_	+*n*_3, 1_	−*n*_2, 1_	−*n*_1, 2_
*n *_4_=	*n *_3, 0_	−*n*_3, 1_	+*n*_2, 2_	+*n*_1, 3_
*n *_5_=	*n *_3, 0_	−*n*_3, 1_	+*n*_2, 2_	−*n*_1, 3_
*n *_6_=	*n *_3, 0_	−*n*_3, 1_	−*n*_2, 2_	+*n*_1, 4_
*n *_7_=	*n *_3, 0_	−*n*_3, 1_	−*n*_2, 2_	−*n*_1, 4_
*n *_*k*,*i*_ ∼	Lap (*λ*/2^3^)	Lap (*λ*/2^3^)	Lap (*λ*/2^2^)	Lap (*λ*/2^1^)

**Table 2:** Gaussian noise injected into wavelet coefficients.

*n *_0_=	*n *_3, 0_	+*n*_3, 1_	+*n*_2, 1_	+*n*_1, 1_
*n *_1_=	*n *_3, 0_	+*n*_3, 1_	+*n*_2, 1_	−*n*_1, 1_
*n *_2_=	*n *_3, 0_	+*n*_3, 1_	−*n*_2, 1_	+*n*_1, 2_
*n *_3_=	*n *_3, 0_	+*n*_3, 1_	−*n*_2, 1_	−*n*_1, 2_
*n *_4_=	*n *_3, 0_	−*n*_3, 1_	+*n*_2, 2_	+*n*_1, 3_
*n *_5_=	*n *_3, 0_	−*n*_3, 1_	+*n*_2, 2_	−*n*_1, 3_
*n *_6_=	*n *_3, 0_	−*n*_3, 1_	−*n*_2, 2_	+*n*_1, 4_
*n *_7_=	*n *_3, 0_	−*n*_3, 1_	−*n*_2, 2_	−*n*_1, 4_
*n *_*k*,*i*_∼	Gauss (3*σ*^2^/4^3^)	Gauss (3*σ*^2^/4^3^)	Gauss (3*σ*^2^/4^2^)	Gauss (3*σ*^2^/4^1^)

**Table 3:** Noise variance and decomposition level *k*.

*k*	Variance of noise	Number of wavelet coefficients
3	3*σ*^2^/4^3^	2^3 − 3^
2	3*σ*^2^/4^2^	2^3 − 2^
1	3*σ*^2^/4^1^	2^3 − 1^

**Table 4:** Max-variance of range query via Haar wavelet and Gaussian mechanism (*l* from 2 to 14).

*l*	*σ * ^2^_sumMax_ (*∗σ*^2^)	*S *_*L*_	*S *_*R*_	*S *_*R*_ − *S*_*L*_ + 1
2	3.000000	0	3	4
3	3.562500	1	6	6
4	4.265625	1	14	14
5	4.910156	3	28	26
6	5.586914	5	58	54
7	6.248291	11	116	106
8	6.917542	21	234	214
9	7.582901	43	468	426
10	8.250217	85	938	854
11	8.916558	171	1876	1706
12	9.583388	341	3754	3414
13	10.249973	683	7508	6826
14	10.916680	1365	15018	13654

**Table 5:** Statistic results of *S*_*L*_ and *S*_*R*_.

*l*	*S *_*L*_		*S *_*R*_	
2	0		3	
3	1	**0** × 2 + 1	6	**3** × 2
4	1	**1** × 2 − 1	14	**6** × 2 + 2
5	3	**1** × 2 + 1	28	**14** × 2
6	5	**3** × 2 − 1	58	**28** × 2 + 2
7	11	**5** × 2 + 1	116	**58** × 2
8	21	**11** × 2 − 1	234	**116** × 2 + 2
9	43	**21** × 2 + 1	468	**234** × 2
10	85	**43** × 2 − 1	938	**468** × 2 + 2
11	171	**85** × 2 + 1	1876	**938** × 2
12	341	**171** × 2 − 1	3754	**1876** × 2 + 2
13	683	**341** × 2 + 1	7508	**3754** × 2
14	1365	**683** × 2 − 1	15018	**7508** × 2 + 2

**Table 6:** Max-variance of range query via Haar wavelet and Gaussian mechanism (any *l*).

*l*	*σ * ^2^_sumMax_ (*∗σ*^2^)	*S *_*L*_	*S *_*R*_	*S *_*R*_ − *S*_*L*_ + 1
2	3.000000	0	3	4
3	3.562500	1	6	6
4	4.265625	1	14	14
5	4.910156	3	28	26
6	5.586914	5	58	54
7	6.248291	11	116	106
8	6.917542	21	234	214
9	7.582901	43	468	426
10	8.250217	85	938	854
11	8.916558	171	1876	1706
12	9.583388	341	3754	3414
13	10.249973	683	7508	6826
14	10.916680	1365	15018	13654
15	11.583327	2731	30036	27306
16	12.250003	5461	60074	54614
17	12.916665	10923	120148	109226
18	13.583334	21845	240298	218454
19	14.250000	43691	480596	436906
20	14.916667	87381	961194	873814
21	15.583333	174763	1922388	1747626
22	16.250000	349525	3844778	3495254
23	16.916667	699051	7689556	6990506
24	17.583333	1398101	15379114	13981014
25	18.250000	2796203	30758228	27962026
26	18.916667	5592405	61516458	55924054
27	19.583333	11184811	123032916	111848106
28	20.250000	22369621	246065834	223696214
29	20.916667	44739243	492131668	447392426
30	21.583333	89478485	984263338	894784854

**Table 7:** Coarse estimation of max-variance for range query.

*n *_0_=	*n *_3, 0_	+*n*_3, 1_	+*n*_2, 1_	+*n*_1, 1_
*n *_1_=	*n *_3, 0_	+*n*_3, 1_	+*n*_2, 1_	−*n*_1, 1_
*n *_2_=	*n *_3, 0_	+*n*_3, 1_	−*n*_2, 1_	+*n*_1, 2_
*n *_3_=	*n *_3, 0_	+*n*_3, 1_	−*n*_2, 1_	−*n*_1, 2_
*n *_4_=	*n *_3, 0_	−*n*_3, 1_	+*n*_2, 2_	+*n*_1, 3_
*n *_5_=	*n *_3, 0_	−*n*_3, 1_	+*n*_2, 2_	−*n*_1, 3_
*n *_6_=	*n *_3, 0_	−*n*_3, 1_	−*n*_2, 2_	+*n*_1, 4_
*n *_7_=	*n *_3, 0_	−*n*_3, 1_	−*n*_2, 2_	−*n*_1, 4_
*n *_*k*,*i*_∼	Gauss (3*σ*^2^/4^3^)	Gauss (3*σ*^2^/4^3^)	Gauss (3*σ*^2^/4^2^)	Gauss (3*σ*^2^/4^1^)
*σ * ^2^_sumMax_=	3*σ*^2^	+3*σ*^2^/4	+3*σ*^2^/2	+3*σ*^2^/2

**Table 8:** *σ* for some *ε* and *δ*.

*ε*	*δ*	*σ*
0.5	0.1	4.4951
0.5	0.01	6.2150
0.5	0.001	7.5530
0.75	0.1	2.9967
0.75	0.01	4.1433
0.75	0.001	5.0353
1.0	0.1	2.2475
1.0	0.01	3.1075
1.0	0.001	3.7765
1.25	0.1	1.7980
1.25	0.01	2.4860
1.25	0.001	3.0212

**Table 9:** Comparison of maximum value between experimental result and theoretical analysis for range 1 to 128.

*ε*	*δ*	Range size	Max-value	*σ * ^2^_sumMax_	Difference
0.5	0.1	106	126.093366	126.252493	−0.159127
0.5	0.01	106	240.611130	241.347894	−0.736764
0.5	0.001	106	358.354318	356.451312	1.903006
0.75	0.1	106	56.957740	56.1109710	0.846769
0.75	0.01	106	109.807594	107.264005	2.543589
0.75	0.001	106	161.471355	158.420708	3.050647
1.0	0.1	106	31.521392	31.5617190	−0.040327
1.0	0.01	106	59.637942	60.336974	−0.699032
1.0	0.001	106	86.007548	89.112828	−3.105280
1.25	0.1	106	20.466279	20.199500	0.266779
1.25	0.01	106	38.351021	38.615663	−0.264642
1.25	0.001	106	57.678236	57.032210	0.646026

## Data Availability

The data used to support the findings of this study are included within the article.
